# Effect of fluralaner administration to domestic dogs on the bloodmeal profile of *Triatoma infestans* in a cluster-randomized trial in the Argentine Chaco

**DOI:** 10.1186/s13071-026-07558-z

**Published:** 2026-07-12

**Authors:** Alejandra Alvedro, María Sol Gaspe, Mateo Rocha-Bazán, Natalia Paula Macchiaverna, Ricardo Esteban Gürtler, Marta Victoria Cardinal

**Affiliations:** 1 Laboratorio de Eco-Epidemiología, Facultad de Ciencias Exactas y Naturales, Universidad de Buenos Aires, Ciudad Universitaria, Buenos Aires, Argentina; 2 Instituto de Ecología, Genética y Evolución de Buenos Aires (IEGEBA), CONICET-Universidad de Buenos Aires, Ciudad Universitaria, Buenos Aires, Argentina; 3 Departamento Agronomía y Ambiente, Facultad de Ingeniería y Ciencias Exactas, Universidad Argentina de la Empresa (UADE), Lima 717, Buenos Aires, Argentina

**Keywords:** *Triatoma infestans*, Xenointoxication, Pyrethroid resistance, Blood-feeding sources

## Abstract

**Background:**

Residual spraying with pyrethroid insecticides remains the main tool in the control of vector-borne transmission of Chagas disease. However, its efficacy has been compromised in regions with pyrethroid-resistant populations of triatomines, as in the Gran Chaco region, where *Triatoma infestans* has developed high levels of resistance. To cope with it, xenointoxication has emerged as a promising strategy. As part of a cluster-randomized trial aimed at evaluating the effects of treating dogs with fluralaner on (peri)domestic infestation and *T. cruzi* infection, we evaluated the effect of fluralaner administration to dogs on the feeding profile of *T. infestans* with particular emphasis on variations in feeding indices on humans, dogs, and chickens.

**Methods:**

We identified bloodmeal sources by enzyme-linked immunosorbent assays on a sample of the *T. infestans* collected before (February 2020) and after (December 2020) the oral administration of fluralaner to dogs in treated clusters. We analyzed 327 triatomines collected pre-intervention and 210 triatomines post-intervention. Peridomestic ecotopes were stratified as of high (HRP) and low risk (LRP) of *T. cruzi* transmission. HRP mainly encompassed kitchens, storerooms, and granaries and the LRP, ecotopes associated with poultry. Only triatomines collected in domiciles and HRP were included for analyses.

**Results:**

In domiciles, humans were the primary bloodmeal source regardless of the intervention arm or time point (> 72%). In HRP, bloodmeals on chickens predominated (> 78%), while dogs (range 0–18%), humans (0–7%), and rodents (1–7%) acted as secondary or marginal sources. No significant effect of treatment arm, intervention time point, and their interaction on the occurrence of bloodmeals on humans, dogs, or chickens was detected via logistic regressions conducted separately for domiciles and HRP. Conversely, dog bloodmeals decreased post-intervention in the treated arm.

**Conclusions:**

Based on these and previously reported results we conclude that we have no evidence to support that the administration of fluralaner to domestic dogs diverts the feeding profile of *T. infestans* to humans at 10 months post-intervention. These findings further support the use of fluralaner in xenointoxication to manage pyrethroid-resistant triatomine populations.

**Graphical Abstract:**

**Figure:**
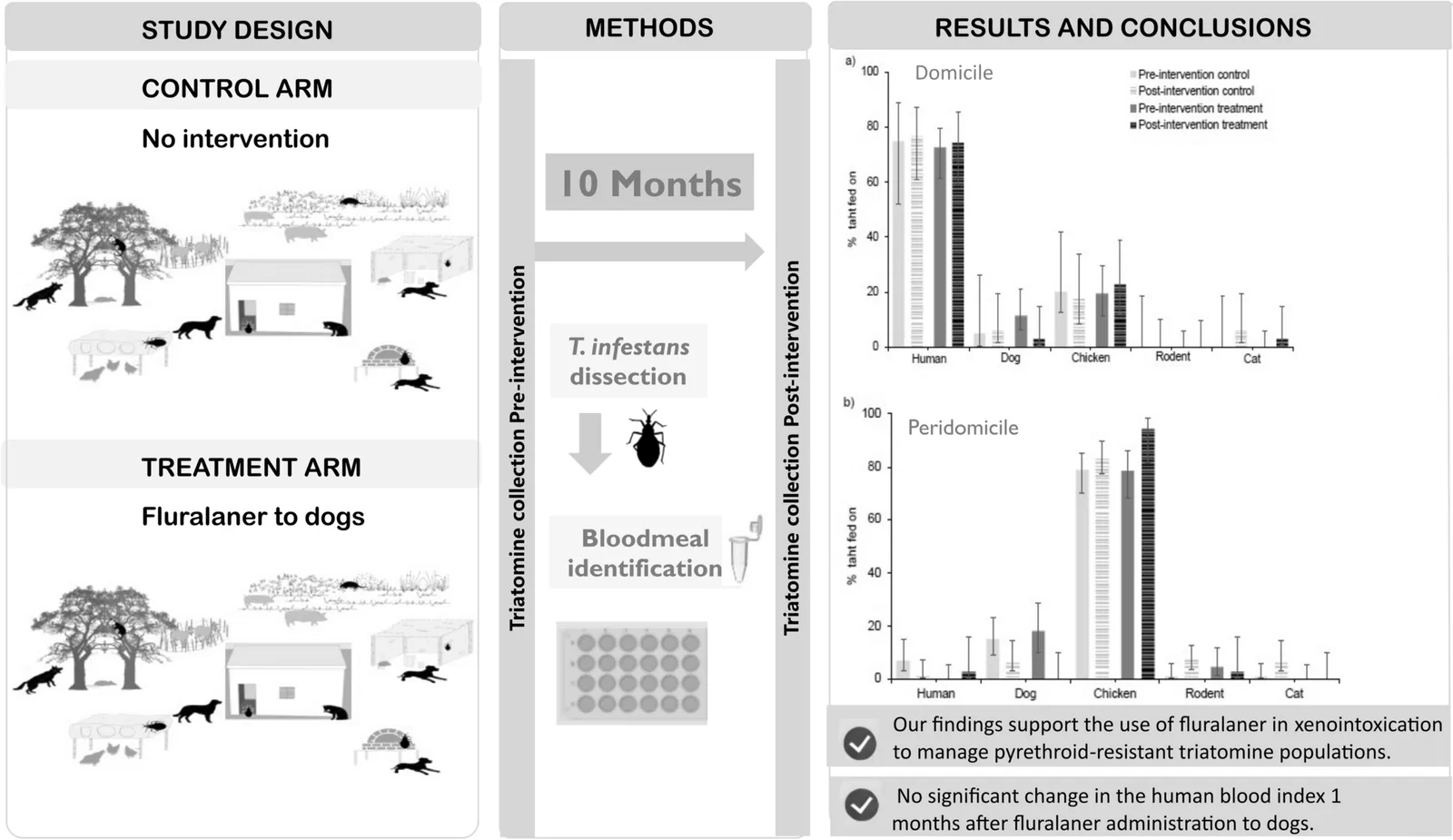

**Supplementary Information:**

The online version contains supplementary material available at https://doi.org/10.1186/s13071-026-07558-z.

## Background

Chemical vector control continues to be the main strategy for controlling a wide range of vector-borne diseases [1]. For Chagas disease, one of the Neglected Tropical Diseases (NTDs) targeted for elimination by 2030, control efforts have primarily relied on pyrethroid insecticides, which generally demonstrated high efficacy [2–4]. Pyrethroid-based multinational initiatives achieved the successful control of domestic vector-borne transmission of *Trypanosoma cruzi* in several South American countries [2, 5]. However, the emergence of pyrethroid resistance in different triatomine species including *Triatoma infestans,* the main domestic vector in the Southern Cone region, threatens control sustainability [6–8].

In this context, integrated vector management incorporating multiple control tactics including environmental management, xenointoxication, and housing improvements is necessary [9–11]. Xenointoxication, the treatment of hosts with systemic insecticides to produce toxic bloodmeals for the vectors, has been evaluated to control triatomines with various compounds and formulations across different animal models, yielding diverse results (e.g., [12–15]). In particular, fluralaner (Bravecto®, MSD Animal), an isoxazoline-class drug, has shown the most promising results in controlling triatomines, both in populations with and without pyrethroid resistance (review in [16]) [12, 14, 15, 17–20]. Treating dogs with an oral dose of fluralaner achieved 90–100% mortality of both susceptible and resistant *T. infestans* for 2 months [17]. Furthermore, a pilot field study demonstrated that treating dogs with fluralaner decreased site-level infestation with *T. infestans*, bug *T. cruzi* infection prevalence and human blood index (HBI, defined as the proportion of triatomines with a detectable bloodmeal that fed on humans) [21, 22], which are components of human transmission risk [23, 24]. In domestic *T. infestans,* blood feeding on humans is deeply modulated by the frequency of dog or chicken-fed triatomines [25, 26]. Therefore, whether fluralaner xenointoxication alters the feeding profile of (peri)domestic *T. infestans* and triatomines in the treated households switch feeding from dogs to humans, which can increase risk of *T. cruzi* transmission to people, are relevant aspects to address in view of preliminary results.

This study is a part of a cluster-randomized trial aimed at evaluating the effects of treating dogs with fluralaner on (peri)domestic infestation and *T. cruzi* transmission. It was carried out in the municipality of Castelli in the Argentinean Chaco, an area where *T. infestans* populations exhibit the highest levels of pyrethroid resistance registered so far [27]. Here, we aim to evaluate the effect of fluralaner administration to dogs on the feeding profile of *T. infestans* with particular emphasis on variations in feeding indices on humans, dogs, and chickens. Given that no repellent effect has been recorded in fluralaner-treated dogs [12], we hypothesized that the administration of fluralaner to domestic dogs does not alter the feeding profile of *T. infestans* found pre-intervention*;* therefore, we particularly expect that the HBI will not increase.

## Methods

### Study area

The field trial was conducted in a rural section of Castelli municipality, Chaco Province, Argentina, a hyperendemic region for Chagas disease with persistent infestations of pyrethroid-resistant *T. infestans* populations [27, 28].

### Study design

The trial included 20 clusters (10–14 houses each) that were randomly assigned to the treated (*n* = 10) or control (*n* = 10) arm (totaling 203 rural households). Each cluster included at least 4 *T. infestans*-infested houses. All houses included in the trial owned at least one dog. In February 2020 (pre-intervention) and December 2020 (post-intervention) house and site infestation with *T. infestans* was assessed using the standard manual search method with a dislodging aerosol (0.2% tetramethrin; Espacial, Buenos Aires, Argentina) [28]. Baseline infestation levels were similar between intervention arms (Gaspe et al., submitted). All eligible dogs (i.e., aged > 2 months with apparent healthy status, and not being pregnant, for females) in the treated arm received a single oral dose of fluralaner (Bravecto®; 25–56 mg/kg body weight) according to the manufacturer's instructions. Householders were informed of the trial goals and effects of fluralaner on ectoparasites, and signed an informed consent form before fluralaner administration. Scheduled activities were abruptly interrupted by the COVID-19 pandemic lockout in March 2020. Consequently, community mobilization workshops to reinforce the importance of exposing treated dogs to infested sites and triatomine surveys to assess short-term treatment effects were canceled.

### Triatomine sampling design

All *T. infestans* collected were identified to developmental stage and sex, and preserved as described [24]. However, pre-intervention triatomines were identified at the field laboratory during field work (which may have allowed some triatomines to consume their bloodmeal contents), whereas triatomines collected post-intervention were immediately frozen. Triatomines collection sites were classified as domiciles and peridomestic ecotopes. Peridomestic ecotopes were further subdivided into high-risk peridomicile (HRP) and low-risk peridomicile (LRP) based on the likelihood of bug infection with *T. cruzi* [29]. HRP mainly include kitchens, storerooms, and granaries, where dogs, cats, and rodents—all competent hosts for *T. cruzi*—are frequently present. LRP refer to sites mainly associated with poultry and other *T. cruzi*-refractory hosts, such as chicken coops and chicken nests. These sites were excluded from our analyses due to their marginal infection prevalence and almost invariant feeding on chickens (> 95%) [22, 29]. All houses infested in domiciles or HRP were included in this study. We randomly selected for examination up to 10 triatomines collected from each infested domiciles and up to 10 from HRP per household in February and December 2020.

### Bloodmeal sources identification

The bloodmeal sources of third-instar nymphs and later stages were identified by means of a direct enzyme-linked immunosorbent assay (ELISA) as described in Gürtler et al. [30], employing antisera against the most frequent (peri)domestic hosts in the study area: dog, cat, chicken, rodent, and human [26]. We considered a bug as reactive when it was positive against any of the antisera tested and as a mixed bloodmeal when it was reactive to at least two different host species [30]. The human blood index (HBI) was calculated as the percentage of ELISA-positive triatomines that had fed on humans, considering single and mixed host bloodmeals.

### Statistical analysis

Agresti-Coull binomial 95% confidence intervals (95% CI) were calculated for the percentage of bloodmeals on each host species. Wilson 95% CI were used when the number of reactive bugs was < 40 or the proportions were near 0 or 1 [31]. Numerical variables (number of hosts per household: humans, dogs, cats, and poultry) were categorized according to their quartiles or medians. Total reactivity between collection strata; and the percentage of houses harboring cats or poultry between treatment arms were evaluated via Fisher’s exact test and medians were compared using the Mann–Whitney U test. Associations between the main observed host blood sources (human, dog, or chicken) in *T. infestans* and selected predictors (intervention arm, time point, and their interaction) were assessed using logistic regression models with random intercepts at the site level or Firth-penalized logistic regressions to account for data structure and the occurrence of complete separation. Although the intervention was randomized at the cluster level, given that a strong decrease in infestation levels at our target sites was registered analyzing at the cluster level was no longer appropriate. Therefore, the collection site was included as a random effect in the models, as it represented the most appropriate grouping scale for individual-level triatomine data. Odds ratios (OR) and their 95% CI were calculated using the “sjPlot” package [32].

Multiple Firth-penalized logistic regressions were implemented in the “logistf” package [33]. Multicollinearity among predictors was assessed using variance inflation factors (VIF) via the “car” package [34]. These analyses were implemented in R (version 2025.09.0, R Foundation for Statistical Computing, Vienna, Austria). Logistic regressions with random effects models were implemented in Stata 15.1 [35].

## Results

Pre-treatment, 47 households were found infested in the treated arm (44%) and 42 in the control arm (46%). Post-treatment, 27 (29%) and 45 (56%) were found infested, respectively (in depth analysis of entomological results are reported elsewhere, Gaspe et al., submitted). In total, 469 *T. infestans* were collected by timed-manual searches in domiciles and HRP ecotopes at pre-intervention (56.3% from the control arm), and 389 were collected in these ecotopes at post-intervention in households that remained infested (67.1% from the control arm) (Table 1). Of these, 327 (50.2% from control houses) and 210 (61.0% from control houses) were examined for bloodmeal sources respectively, with 75.5% and 86.2% showing reactivity (percentage of ELISA-reactive bugs) to at least one of the antisera tested (Table 1). Total reactivity was significantly higher in triatomines collected post-intervention (Fisher’s exact test, *df* = 1, *P* = 0.002). Very few reactive *T. infestans* collected post-intervention fed on at least two different host species (3.8%).

**Table 1:** Distribution of *T. infestans* collected*,* examined, and reactive for bloodmeal sources by intervention time point, treatment, and ecotope; Castelli, Chaco Province, February–December 2020

**Time point and treatment arm**	**Ecotope**	**% of triatomines**		
		**Examined (No. collected)**	**Reactive (No. examined)**	**With mixed bloodmeals (No. reactive)**
**Pre-intervention**				
Treatment	Domicile	98.5 (67)	93.9 (66)	3.2 (62)
	HRP	70.3 (138)	67.0 (97)	4.6 (65)
	Subtotal	79.5 (205)	77.9 (163)	3.9 (127)
Control	Domicile	56.0 (50)	71.4 (28)	0.0 (20)
	HRP	63.5 (214)	73.5 (136)	3.0 (100)
	Subtotal	62.1 (264)	71.6 (164)	2.5 (120)
Total		69.7 (469)	75.5 (327)	3.2 (247)
Post-intervention				
Treatment	Domicile	50.6 (75)	92.1 (38)	2.8 (35)
	HRP	83.1(53)	79.5 (44)	0.0 (35)
	Subtotal	64.1 (128)	85.4 (82)	1.4 (70)
Control	Domicile	85.4 (48)	82.9 (41)	5.8 (34)
	HRP	40.8 (213)	88.5 (87)	5.1 (77)
	Subtotal	49.1 (261)	86.7 (128)	5.4 (111)
Total		53.9 (389)	86.2 (210)	3.8 (181)

HRP: High-risk peridomicile

Overall, each house harbored a median of 3–4 people, 4 dogs, 1 cat, and 30 chickens–ducks. Treated and control houses harbored similar frequencies and median abundances of humans, dogs, and poultry (Additional file 1, Table S1). We found no differences in the reported frequencies of dogs, cats, and poultry resting or roosting inside domiciles between the treated or control arms (Fisher’s exact test: *df* = 1, *P* > 0.05 in all cases) (Additional file 1, Table S1).

**Fig. 1:**
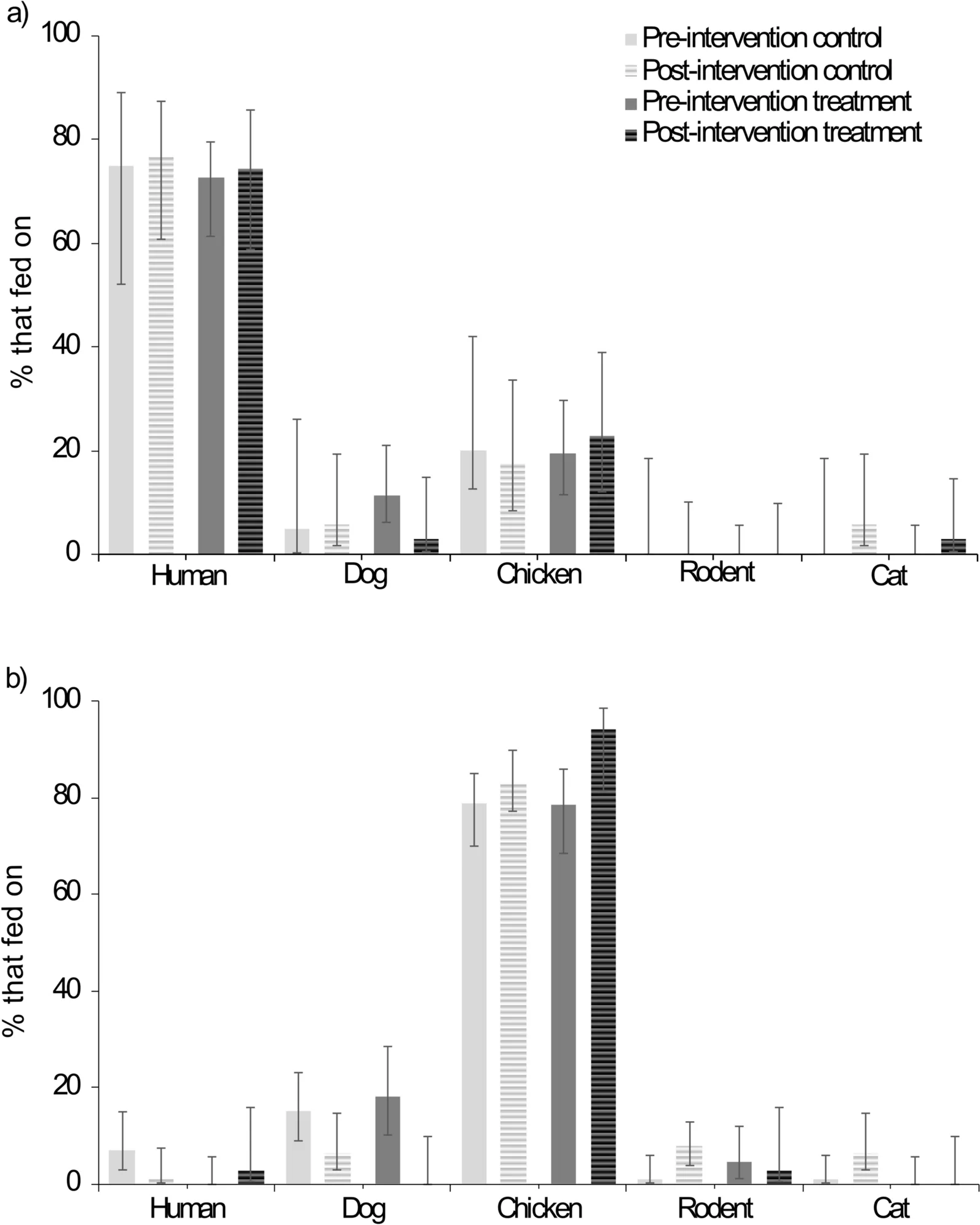
Distribution of bloodmeal sources in *T. infestans* by intervention time point, treatment and ecotope category: **a** domiciles and **b** high-risk peridomiciles. Whiskers show Agresti–Coull and Wilson 95% confidence intervals

The effects of fluralaner treatment on host bloodmeals were assessed separately for each ecotope category (Fig. 1). In domiciles, humans were the primary bloodmeal source regardless of the intervention arm or time point (> 72% in all cases), with the highest HBI observed in the control group pre-intervention (75.0%) (Fig. 1a). Chickens (range 5.9–22.9%) and dogs (range 2.9–11.3%) were secondary bloodmeal sources, detected in both time points and arms, while bloodmeals from cats were only detected post-intervention (5.9% and 2.9%, control and treated group, respectively). A non-significant decrease in dog bloodmeals from 11 to 3% in the treated group after fluralaner administration was observed (Fisher's exact test *df* = 1, *P* = 0.25), while no changes were observed in the control group (Fisher's exact test *df* = 1, *P* = 0.89).

In the HRP the diversity of bloodmeal sources was maximal (i.e., all tested hosts acted as bloodmeal sources), with feeding on rodents detected both before and after the intervention (Fig. 1b). In these ecotopes, chickens were the primary bloodmeal source detected across both time points and arms (> 80%). No bloodmeal on dogs was detected post-intervention in the treated arm.

No significant effect of treatment arm, intervention time point, and their interaction on the occurrence of bloodmeals on humans, dogs, or chickens was detected via random effects or Firth-penalized, logistic regressions conducted separately for domiciles and HRP (Table 2).

**Table 2:** Multiple regression analysis for the occurrence of bloodmeals on humans, dogs, and chickens in *Triatoma infestans* collected from domiciles and HRP in Castelli, Chaco.

E**cotope explanatory variables**	**Levels**	**Human bloodmea**l			**Dog bloodmeal**			C**hicken bloodmeal**		
		**Odds ratios**	**95% CI**	***P-value***	**Odds ratios**	**95% CI**	***P-value***	**Odds ratios**	**95% CI**	***P-value***
Domicile										
Time point	Pre-intervention	Reference								
	Post-intervention	1.42	0.04–55.44	0.85	1	0.12–8.17	1.00	0.84	0.22–3.21	0.79
Intervention	Control	Reference								
	Treatment	1.34	0.05–35.22	0.86	1.76	0.28–10.92	0.52	0.91	0.27–3.05	0.87
Time point × Intervention	Pre-intervention × Control	Reference								
	Post-intervention × Treatment	0.05	0.00–27.42	0.36	0.32	0.02–5.13	0.41	1.49	0.28–7.93	0.64
HRP										
Time point	Pre-intervention	Reference								
	Post-intervention	0.24	0.04–1.45	0.08	0.42	0.15–1.16	0.08	0.39	0.04–3.49	0.40
Intervention	Control	Reference								
	Treatment	0.10	0.01–1.70	0.03	1.42	0.63–3.18	0.39	0.41	0.05–3.43	0.41
Time point × Intervention	Pre-intervention × Control	Reference								
	Post-intervention × Treatment	23.3	0.58–928.30	0.07	0.13	0.01–2.71	0.11	10.95	0.22–540.45	0.23

Logistic regressions with random effects were employed for human bloodmeals in domiciles and chicken bloodmeals in HRP; Firth-penalized logistic regressions were employed for dog and chicken bloodmeals in domiciles and human and dog bloodmeals in HRP

HRP: high-risk peridomicile

## Discussion

Our findings show that the administration of fluralaner to dogs did not significantly modify the human blood index at 10 MPI (months post-intervention), with human–vector contact remaining similar between control and treated arms based on the ELISA-reactive triatomines. In addition, we found no significant effect of treatment on the occurrence of bloodmeals on dogs or chickens both in domiciles and HRP via Firth-penalized or random effects logistic regressions.

Dog-targeted strategies, such as deltamethrin impregnated collars, may exert a repellent effect on triatomines, which could potentially divert their feeding toward humans [36]. Whether fluralaner prevents or not biting from blood-sucking vectors or diverts their bloodmeals to humans are key features for preventing the transmission of pathogens. The lethal effects of fluralaner have been studied across different arthropod vectors, including ticks, triatomines, mosquitoes, fleas, and sandflies, and several mammal hosts [37–39] (reviewed in Jiang and Old [16]). In triatomines, fluralaner did not modify host-seeking behavior or blood-engorgement success [12, 17, 19]. The lack of a repellent effect allows triatomines to feed on treated animals and ingest a lethal dose of fluralaner, resulting in very high (> 80%) short-term mortality [15, 17, 18]. This is consistent with our results, where no significant effect of fluralaner administration on the frequency of blood feeding on humans was detected 10 MPI.

The feeding profile registered in the present study is consistent with previous results from a small field trial on the effects of fluralaner administration to dogs [22]. There, a re-treatment with fluralaner was administered at 7 months after the first dose. In both studies, humans were the primary bloodmeal source in domiciles, whereas chickens predominated in peridomestic sites. At 12 months after the last administration of fluralaner to dogs, human, and chicken bloodmeals remained at baseline levels, while nil or low bloodmeals on dogs were detected, as identified in this study 10 MPI. The absence of dog bloodmeals in the treated arm could be explained by the death of any triatomines feeding on fluralaner-treated dogs. The few dog bloodmeals at 10 MPI may represent a subset of surviving triatomines that fed on newly acquired dogs or on a few untreated dogs. Considering these results, a multi-host fluralaner-based xenointoxication strategy involving dogs, cats, and chickens could further enhance the suppression of residual infestations and overcame the limitations of single-host interventions [15, 20].

This study has some limitations. Our scheduled activities were abruptly interrupted by the government-imposed lockdown related to the COVID-19 pandemic in March 2020 in Argentina. This altered our plans in two relevant aspects; first, we were unable of deploying community-engaging workshops to explain the importance of exposing the treated dogs to the target sites. In the second place, assessment of short-term treatment effects was impossible. Third, the December 2020 survey was remotely supervised by the research team and triatomines were frozen the same day of collection. This may explained the higher reactivity recorded in post-intervention bugs. Additionally, ELISA-based feeding profile analyses may underestimate the proportion of mixed bloodmeals, as this approach may fail to detect degraded meals, samples with little blood content or host species outside the panel of antisera tested in comparison with metabarcoding or NGS-based techniques. These approaches may improve the sensitivity of bloodmeal test and better characterize host-feeding diversity in triatomine populations, particularly for intrusive or sylvatic triatomine species [40].

## Conclusions

we have no evidence to support that the administration of fluralaner to domestic dogs modifies the feeding profile of *T. infestans* on human, dogs, or chickens at 10 MPI based on the ELISA-reactive triatomines. No increase of the HBI was detected here and previously [22] giving further support to the use of fluralaner xenointoxication within an integrated vector management strategy to address persistent house infestation with both susceptible and pyrethroid-resistant triatomines.

## Supplementary Information

Additional file 1. Supplementary table. Frequency of main bloodmeal hosts, median abundance per house, and reported indoor resting/roosting frequency in control and treated houses; Castelli, Chaco Province, February 2020

Additional file 2. Supplementary table. Database

## Data Availability

All data generated or analyzed during this study are included in this published article and its additional files.

## Abbreviations

COVID-19: Coronavirus Disease 2019
ELISA: Enzyme-Linked ImmunoSorbent Assay
HBI: Human blood index
HRP: High-risk peridomicile
LRP: Low-risk peridomicile
MPI: Months post-intervention
