# Supplementary material for: Effect of fluralaner administration to domestic dogs on the bloodmeal profile of Triatoma infestans in a cluster-randomized trial in the Argentine Chaco
Source: Parasit Vectors. 2026 Jul 12;19:432. doi: 10.1186/s13071-026-07558-z (PMC13640203; doi:10.1186/s13071-026-07558-z)
Supplement: Supplementary file 1 [file 13071_2026_7558_MOESM1_ESM.docx]

**Additional file 1. S**upplementary table. Frequency of main bloodmeal hosts, median abundance per house (first and third quartiles, Q1-Q3) and reported indoor resting/roosting frequency in control and treated houses; Castelli, Chaco Province, February 2020.

| Trial arm | Median number per household (Q1, Q3) | | | | % of houses harboring (No. with data)* | | % of household with host indoors (No. with data)# | | |
| --- | --- | --- | --- | --- | --- | --- | --- | --- | --- |
| (No. of houses) | Humans | Dogs | Cats | Poultry | Cats | Poultry | Dogs | Cats | Poultry |
| Control (30) | 3 (2-4) | 4 (3-6) | 1 (0-1) | 30 (2-46) | 62 (29) | 100 (29) | 63 (8) | 47 (15) | 0 (20) |
| Treatment (32) | 3 (2-4) | 5 (3-5) | 1 (0-2) | 30 (10-50) | 58 (31) | 87 (31) | 90 (10) | 35 (17) | 13 (24) |

*All houses harbored dogs, as it was a prerequisite for participating in the trial.

\# The variable "% of households with hosts indoors" refers to the proportion of households where the specific host sleeps or is allowed to rest indoors.
